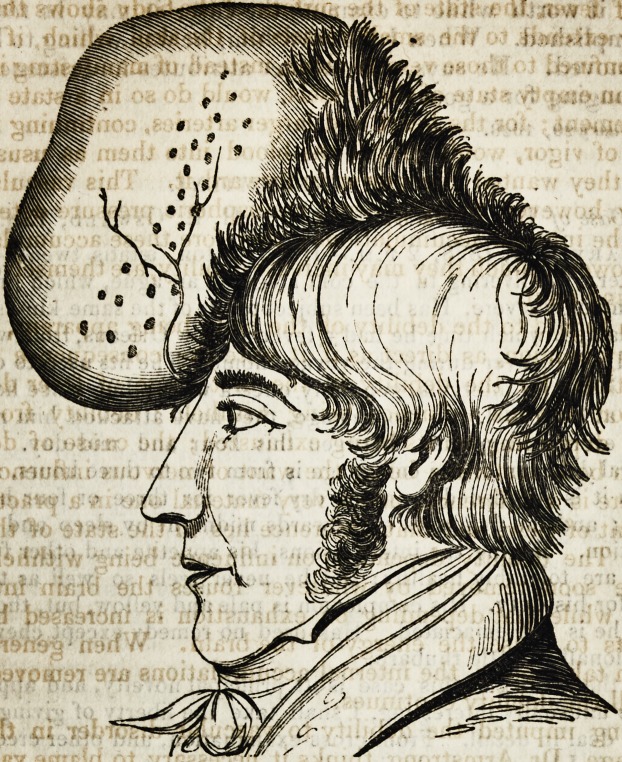# Case of Large Tumor on the Forehead

**Published:** 1828-04

**Authors:** Henry Thompson


TUMOR.

*Case of large Tumor on the Forehead.*
By Henry Thompson, m.d.

James Lipscombe, aged forty-six, a waggoner, has had a tumor on the forehead from his birth, which has never given him pain: at first it was the size of a nut, and has not, he says, increased very materially until within the last six years, nor did he require a hat of more than the usual dimensions. Within the last-mentioned period, the tumor has increased to a very distressing degree, causing him much pain in the neck from its weight, and has attained the following dimensions: —two feet four inches round the base, three feet round the widest part, and two feet four inches across. When I last saw the patient, about six months ago, the tumor appeared to contain fluid in some parts of it. On inquiry, I find he is still living, and continues to come twice a week to the Talbot Inn in the Borough:* should any of your readers, therefore,

* I am not quite sure of the name of the inn, but think it is the Talbot.

think it worth while to examine the case, it may be readily accomplished. When I last saw him, he was willing to have it removed. He says, his mother attributes its existence to her longing for a goose's egg.

*February* 20, 1828.

## Figures and Tables

**Figure f1:**